# Dulaglutide and Dapagliflozin Combination Concurrently Improves the Endothelial Glycocalyx and Vascular and Myocardial Function in Patients with T2DM and Albuminuria vs. DPP-4i

**DOI:** 10.3390/jcm13247497

**Published:** 2024-12-10

**Authors:** Emmanouil Korakas, John Thymis, Evangelos Oikonomou, Konstantinos Mourouzis, Aikaterini Kountouri, Loukia Pliouta, Sotirios Pililis, George Pavlidis, Stamatios Lampsas, Konstantinos Katogiannis, Lina Palaiodimou, Georgios Tsivgoulis, Gerasimos Siasos, Ignatios Ikonomidis, Athanasios Raptis, Vaia Lambadiari

**Affiliations:** 1 2nd Department of Internal Medicine Research Unit and Diabetes Centre, Attikon Hospital, Medical School, National and Kapodistrian University of Athens, Rimini 1 Str., Chaidari, 12462 Athens, Greece; mankor-th@hotmail.com (E.K.); katerinak90@hotmail.com (A.K.); plioutaloukia@gmail.com (L.P.); sotiris181@yahoo.gr (S.P.); geo_pavlidis@yahoo.gr (G.P.); lampsas.stam@gmail.com (S.L.); atraptis@med.uoa.gr (A.R.); 2 2nd Department of Cardiology Laboratory of Preventive Cardiology and Echocardiography Department, Attikon Hospital, Medical School, National and Kapodistrian University of Athens, 12462 Athens, Greece; johnythg@gmail.com (J.T.); kenndj89@gmail.com (K.K.); ignoik@gmail.com (I.I.); 3 3rd Department of Cardiology, Medical School, Sotiria Chest Disease Hospital, National and Kapodistrian University of Athens, 11527 Athens, Greece; boikono@gmail.com (E.O.); mourouzis2005@yahoo.gr (K.M.); 4 2nd Department of Neurology, “Attikon” University Hospital, School of Medicine, National and Kapodistrian University of Athens, Rimini 1, Chaidari, 12462 Athens, Greecetsivgoulisgiorg@yahoo.gr (G.T.); 5 Cardiovascular Division, Harvard Medical School, Brigham and Women’s Hospital, Boston, MA 02115, USA; ger_sias@hotmail.com

**Keywords:** diabetes mellitus, albuminuria, arterial stiffness, endothelial glycocalyx, global longitudinal strain

## Abstract

**Background**: The association between diabetic nephropathy and arterial elasticity and endothelial function is well established. In this study, we compared the effect of the combination of dulaglutide and dapagliflozin versus DPP-4 inhibitors on the endothelial glycocalyx, arterial stiffness, myocardial function, and albuminuria. **Methods**: Overall, 60 patients were randomized to combined dulaglutide and dapagliflozin treatment (n = 30) or DPP-4 inhibitors (DPP-4i, n = 30) (ClinicalTrials.gov: NCT06611904). We measured at baseline and 4 and 12 months post-treatment: (i) the perfused boundary region of the sublingual arterial microvessels, (ii) pulse wave velocity (PWV) and central systolic blood pressure (cSBP), (iii) global left ventricular longitudinal strain (GLS), and (iv) urine albumin-to-creatinine ratio (UACR). **Results**: After twelve months, dual therapy showed greater improvements vs. DPP-4i in PBR (2.10 ± 0.31 to 1.93 ± 0.23 μm vs. 2.11 ± 0.31 to 2.08 ± 0.28 μm, *p* < 0.001), UACR (326 ± 61 to 142 ± 47 mg/g vs. 345 ± 48 to 306 ± 60 mg/g, *p* < 0.01), and PWV (11.77 ± 2.37 to 10.7 ± 2.29 m/s vs. 10.64 ± 2.44 to 10.54 ± 2.84 m/s, *p* < 0.001), while only dual therapy showed improvement in cSBP (130.21 ± 17.23 to 123.36 ± 18.42 mmHg). These effects were independent of glycemic control. Both treatments improved GLS, but the effect of dual therapy was significantly higher compared to DPP-4i (18.19% vs. 6.01%, respectively). **Conclusions**: Twelve-month treatment with dulaglutide and dapagliflozin showed a greater improvement in arterial stiffness, endothelial function, myocardial function, and albuminuria than DPP-4is. Early initiation of combined therapy as an add-on to metformin should be considered in these patients.

## 1. Introduction

The prevalence of type 2 diabetes mellitus (T2DM) is rapidly increasing worldwide and, as a result, macro- and microvascular complications pose an ever-increasing health and socioeconomic burden. Apart from exacerbating atherosclerosis and cardiac remodeling, T2DM leads to adverse cardiovascular events such as heart failure and diabetic nephropathy (DN), which is also the leading cause of chronic kidney disease (CKD) [1]. Numerous large-scale RCTs have demonstrated substantial beneficial effects of glucagon-like peptide-1 receptor agonists (GLP-1RAs) and sodium–glucose cotransporter-2 inhibitors (SGLT-2is) on the risk of cardiovascular complications, such as myocardial infarction and stroke, heart failure, and the progression of CKD to end-stage renal disease (ESKD) and albuminuria [2]. Apart from their hypoglycemic actions, these drugs exert beneficial anti-inflammatory, anti-oxidative, and anti-fibrotic effects. However, data regarding their effects on the endothelial glycocalyx, arterial stiffness, and myocardial function are still relatively scarce [3].

The endothelial glycocalyx is a mesh of glycoproteins, proteoglycans, and glycosaminoglycans that covers the endothelium and has a vital role in permeability, mechanotransduction, and other functions like immunity and hemostasis [4]. Changes in the glomerular basement membrane (GBM) and podocytes are typical in the pathogenesis of diabetic kidney disease (DKD); however, proteinuria has also been noted even in the absence of such structural changes, implying that changes in glycocalyx integrity and, eventually, the endothelium are a key factor in the natural history of DKD [5]. Both human and animal models have shown a negative association between glycocalyx integrity and hyperglycemia [6]. Novel, fast, and non-invasive techniques facilitate the assessment of the glycocalyx in the sublingual microvessels, obtaining estimates that are considered representative of the systemic vasculature [7].

Arterial stiffness, as assessed by pulse wave velocity, has an independent association with the macro- and microvascular complications of T2DM [8]. Also, some studies have demonstrated a negative correlation between arterial stiffness and estimated glomerular filtration rate (eGFR), implying its possible role as a predictive biomarker in CKD progression [9].

However, the synergistic effect of GLP-1RAs and SGLT-2is on subclinical markers of endothelial, vascular, and cardiac function in patients with T2DM has yet to be extensively studied. In addition, data on the correlation of such indices of subclinical disease with renal function are scarce. In general, it is known that GLP-1RAs, apart from enhancing insulin sensitivity, exert anti-inflammatory actions by downregulating pro-inflammatory cytokines and chemokines like IL-6, IL-8, TNF-α, and IL-21 and alleviating oxidative stress [10]. In a similar way, SGLT-2is have immunomodulatory capacities, which include decreasing reactive oxygen species (ROS) production, increasing mitophagy, and downregulating NF-kB signaling, among others, thus contributing to the reversal of cardiac remodeling and substantial cardiorenal benefits [11]. In a previous study by Ikonomidis et al. [11], the combination of liraglutide and empagliflozin decreased PBR and PWV by 9.1% and 13%, respectively, after a year of treatment, in patients with T2DM and high or very high cardiovascular risk. Data regarding the combined treatment are scarce since dulaglutide was studied separately in a report by Tuttolomondo et al. that noted a reduction in PWV by 4% after 9 months of treatment. In addition, DPP-4 inhibitors (DPP-4i) have been a first-line choice in the Greek population over the last decade, despite the neutral cardiovascular and renal results from large-scale RCTs [12]. It is interesting though that, with the exception of large-scale RCTs, renal function and albuminuria are not usually among the primary or secondary endpoints of investigator-driven studies. In addition, data on the correlation between renal function and markers of endothelial dysfunction are limited; the endothelial glycocalyx, in particular, cannot readily be measured in the majority of healthcare centers. In this study, in patients with T2DM and albuminuria, we hypothesized that the endothelial glycocalyx, arterial stiffness, LV myocardial deformation, and albuminuria would be improved after treatment with the combination of dulaglutide and dapagliflozin as an add-on treatment to metformin compared with DPP-4i.

## 2. Materials and Methods

### 2.1. Study Population

We examined 60 consecutive subjects with T2DM (male-to-female ratio: 48:12; mean age: 61 years) and albuminuria. The inclusion criteria were history of T2DM, age 18–75 years old, HbA1c 7–10%, the presence of albuminuria, and eGFR > 60 mL/min. All patients underwent a clinical, vascular, and echocardiography examination at 4 and 12 months after inclusion in the study. Patients were recruited from the cardiometabolic outpatient clinic of Attikon Hospital, and they were randomized to receive, as an add-on treatment to metformin, either dulaglutide (0.75 mg once weekly for the first month and 1.5 mg once weekly afterward) and 10 mg oral dapagliflozin once daily or any DPP-4i (100 mg oral sitagliptin daily, 100 mg oral vildagliptin daily, 25 mg oral alogliptin daily, or 5 mg oral linagliptin daily) for 12 months.

Exclusion criteria included: active infection, eGFR < 60 mL/min/1.73 m^2^, severe hepatic impairment, history of malignancy within the last 5 years, cardiovascular events within the recent month, any major surgery or severe trauma over the last 3 months and chronic inflammatory or autoimmune diseases. None of the female patients received hormone replacement treatment. All patients were treated with metformin and a stable (at least for 3 months) maximally tolerated dose of angiotensin-converting enzyme inhibitors (ACEis) or angiotensin 2 receptor blockers (ARBs) at baseline.

The investigation conforms to the principles outlined by the Declaration of Helsinki. The study was approved by the Ethics Committee of Attikon Hospital (456/11-06-2017). Written informed consent was obtained before patients’ participation.

### 2.2. Blood Pressure Measurement

Each patient rested in the supine position for 10 min in a quiet room at 23 °C. Brachial blood pressure was measured using an automated digital oscillometric sphygmomanometer (TensioMed, Budapest, Hungary). Two sequential measurements separated by 2-min intervals were obtained, and the mean value was used for statistical analysis.

### 2.3. Endothelial Glycocalyx

The perfused boundary region (PBR) of the sublingual arterial microvessels with a diameter that ranged from 5 to 25 μm was measured via Side stream Dark Field imaging (Microscan, Glycocheck, Microvascular Health Solutions Inc., Salt Lake City, UT, USA).

The PBR is the cell-poor layer that results from the separation between the red blood cell column and plasma on the luminal surface. An increased PBR value is a marker of reduced glycocalyx thickness.

### 2.4. Arterial Stiffness and Central Hemodynamics

We measured carotid–femoral PWV and central aortic pressures (central systolic and diastolic) using a tonometry device by Complior (Alam Medical, Vincennes, France). Normal values were PWV < 10 m/s.

### 2.5. LV Myocardial Deformation

Studies were performed using a Vivid (GE Medical Systems, Horten, Norway) ultrasound system and digitally stored in a computerized station (EchoPac GE202, Horten, Norway). All studies were analyzed by 2 observers, blinded to clinical and laboratory data. We measured LV global longitudinal strain (GLS; %) from 2-dimensional echocardiography images from the apical 4-, 2-, and 3-chamber views using a 17 LV segment model (EchoPacPC 203; GE Healthcare, Horten, Norway). The normal value for GLS is considered to be −22.5 ± 2.7%.

### 2.6. Statistical Analysis

Statistical analysis was conducted using the Statistical Package for Social Sciences (IBM SPSS Statistics for Windows, Version 26.0. Armonk, NY, USA). Continuous variables were expressed either as the mean ± standard deviation in the case of normal distribution or as the median and interquartile range if non-normally distributed. Normality testing was performed with the Kolmogorov–Smirnov test. Differences in continuous variables were evaluated using the Student’s *t*-test and Mann–Whitney U test, as appropriate. Only diabetes duration did not follow a normal distribution, and therefore, we used the Mann–Whitney U test. Categorical variables were presented as absolute and relative frequencies and were analyzed by performing the chi-square test or Fisher’s exact test. All analyses were intention to treat. Analysis of variance (ANOVA) for repeated measurements was performed for (a) measurements of the examined markers at baseline and at 4 and at 12 months of treatment and (b) the effects of different treatments, as a between-subject factor (dulaglutide and dapagliflozin vs. DPP-4i). The F- and *p*-values of the interaction between the time of measurement of the examined markers and the type of treatment were calculated. Moreover, the F- and the corresponding *p*-values of the comparison between treatments were estimated. All statistical tests were two-tailed and a *p* < 0.05 was considered statistically significant.

## 3. Results

### 3.1. Baseline Characteristics

The baseline characteristics of the study population are shown in Table 1. All patients had similar ages, cardiovascular medications, glycosylated hemoglobin (HbA1c), weight, and BMI at inclusion.

### 3.2. Metabolic Control

All patients had significantly improved plasma glucose levels and HbA1c at 4 months and 12 months (*p* < 0.001). BMI was decreased in the overall population after 4 and 12 months (*p* < 0.001). However, there was a significant interaction between the type of treatment and the change in BMI post treatment (F = 14.34, *p* for interaction < 0.001). Patients treated with the dulaglutide and dapagliflozin combination showed a reduction in BMI at 4 and 12 months (*p* < 0.05 for all changes), whereas the DPP-4i group showed no significant reduction in BMI at any study timepoint (Table 2).

### 3.3. Renal Function and Albuminuria

All patients showed a reduced urine albumin-to-creatinine ratio (UACR) at the end of the study period (*p* < 0.001). There was significant interaction between the type of treatment and the change in UACR (F = 30.77, *p* for interaction < 0.001). After 4 months, patients in the combination group showed a higher reduction in UACR compared to the DPP-4i group (−33.7% vs. −11.4%, respectively, *p* < 0.05). After 12 months, patients in the combination group once again showed a higher reduction in UACR compared to the DPP-4i group (−52.3% vs. −14.5%, respectively, *p* < 0.01). Similarly, eGFR was slightly improved in the combination group, while no effect was observed in the DPP-4i group (Table 3).

### 3.4. Endothelial Glycocalyx, Arterial Stiffness, and Central Hemodynamics

There was significant interaction between the type of treatment and the change in PBR (F = 5.85, *p* for interaction = 0.005). At 4 months, no change in PBR was observed in all patients (*p* > 0.05). Conversely, at 12 months, all patients showed a reduced PBR (*p* < 0.01) with the combination of dulaglutide and dapagliflozin (−8.1%), while the patients in the DPP-4i group showed a non-significant decrease.

Significant interaction between the type of treatment and the change in PWV and central SBP was observed (F = 4.04 [*p* for interaction = 0.023], F = 3.1 [*p* = 0.048], respectively). At 4 months, the combination of dulaglutide and dapagliflozin reduced PWV by −7.48%, an effect which was augmented at 12 months (−9.1%), compared to no effect in the DPP-4i group. Similarly, central SBP was reduced only in the combination group, an effect, however, that was evident only after 12 months of treatment (Table 4).

### 3.5. Changes in LV Myocardial Deformation

All patients showed statistically significant improvements in GLS and PWV/GLS ratio at the end of the study period, though not after the first 4 months. The improvement was statistically significant in both groups; however, patients under the combination of dulaglutide and dapagliflozin showed an almost three-fold greater improvement compared to the DPP-4i group (Table 5).

### 3.6. Correlations Between Albuminuria, Metabolic, Endothelial, Vascular, and LV Function Markers

In the entire study population, the change in UACR was associated with the change in PWV/GLS ratio (r = 0.41, *p* = 0.015), the change in PWV (r = 0.3, *p* = 0.01), and the change in PBR (r = 0.15, *p* = 0.026).

## 4. Discussion

In this study, we showed that the combination of dulaglutide and dapagliflozin led to a more profound reduction in BMI, cSBP, arterial stiffness, endothelial glycocalyx thickness, albuminuria, and GLS compared to DPP-4i, in patients with T2DM and proteinuria, despite similar benefits in terms of glycemic control.

In terms of arterial stiffness, the benefit of combined treatment was evident even after the first 4 months of treatment, and it was sustained and augmented after 12 months. These findings are in accordance with the majority of the literature data. Regarding dulaglutide, a study by Tuttolomondo et al. [12] showed a reduction of −4% after 9 months of treatment, concurrently with a reduction in blood pressure. As in our study, benefits were clear from the first 3 months of treatment. Xie et al. [15] showed that dulaglutide, as a second-line treatment after metformin, reduced PWV by −22% after 3 months, an effect that was associated with respective reductions in inflammatory markers and increases in endothelial progenitor cells (EPCs). Notably, dulaglutide has not been thoroughly studied, and most of the available data refer to liraglutide, either as a monotherapy or in combination with empagliflozin. On the other hand, the association between SGLT-2i and arterial stiffness is better established, albeit contradictions still exist. In a previous study by our group [12], apart from its combination with liraglutide, empagliflozin per se reduced PWV by 10.1% after 12 months. Solini et al. [16] showed a reduction in PWV with dapagliflozin (10.1 ± 1.4 vs. 8.9 ± 1.6 m/s, *p* < 0.05), an effect which was exerted after only 2 days of treatment. Similar to our results, blood pressure was also reduced, which is indicative of the rapid hemodynamic actions of dapagliflozin; surprisingly, however, cSBP showed a non-significant reduction [16]. In obese people and those with T2DM, without established cardiovascular disease, dapagliflozin also decreased PWV, though it must be noted that no comparator group existed [17]. In the largest randomized trial to date, Papadopoulou et al. [18] showed profound reductions in PWV (−0.16 ± 0.32 vs. 0.02 ± 0.27 m/s, *p* = 0.007) after 12 weeks of treatment, with simultaneous improvement in SBP, DBP, and cSBP. On the other hand, reports by Karalliede et al. [19] and Patoulias et al. [20] showed no effect of SGLT-2i on arterial stiffness, and a recent meta-analysis did not confirm any beneficial actions either, with the reasons behind these discrepancies still being vague. As for DPP-4is, the lack of benefit in our study is in accordance with some reports, where the comparator drug was glibenclamide, regardless of the specific DPP-4i used [21,22,23]. A recent meta-analysis showed a rather beneficial effect, but the high degree of heterogeneity among the studies does not allow for robust conclusions to be drawn [24].

The integrity of the endothelial glycocalyx reflects endothelial function. PBR, an estimate of glycocalyx thickness, was reduced from 2.10 μm to 1.93 μm after 12 months of combination treatment, with the beneficial effects being pronounced from the first 4 months, contrary to the neutral effects in the DPP-4i group. These findings are considered novel, as there is a paucity of data regarding the effect of commercially available treatments on glycocalyx integrity. The only available study is by Ikonomidis et al. [12], where the liraglutide and empagliflozin combination reduced PBR after 12 months. In addition, PBR is not a commonly used glycocalyx marker; endothelial function is usually evaluated through flow-mediated dilation (FMD). Results with dapagliflozin are conflicting, an effect which might be attributed to the fact that in most studies, FMD levels were near normal [25,26,27]. In the meta-analysis by Batzias et al., SGLT-2i seemed to have an overall positive effect on FMD, contrary to GLP-1RAs and DPP-4i [28].

Combined treatment with dulaglutide and dapagliflozin significantly reduced UACR after 4 months, an effect that was sustained and magnified after 12 months. A similar reduction was also noted in the DPP-4i group; however, the magnitude of reduction was about three times lower. These results are expected based on the available study data. The DAPA-CKD trial showed significant improvements in the composite renal endpoint [29], and in the DELIGHT trial, dapagliflozin reduced UACR by 21% [30]. In a sub-study of the REWIND trial [31], dulaglutide reduced the risk for the renal endpoint by 15%, mainly affecting the establishment of macroalbuminuria, in agreement with other large RCTs regarding GLP-1RAs. In the meta-analysis by Tuttle et al. [32], dulaglutide did not confer significant benefits to creatinine levels or eGFR, but it decreased proteinuria to a significant level. On the other hand, linagliptin can have beneficial (CARMELINA study) [33] or neutral (MARLINA-T2D study) [34] effects on UACR, regardless of its potent hypoglycemic effect. In large-scale meta-analyses, DPP-4is have been associated with greater eGFR reduction compared to other agents, but with an 11–12% lower risk of establishment of new-onset proteinuria and significantly higher proteinuria regression (RR: 1.22, 95% CI: 1.10–1.35) [35,36]. It is interesting that, despite the overmasculinization in our study, the results were consistent in both sexes, contrary to recent data stating that sex modifies the association between UACR and diabetes among adults [37]. The fact that, in our study, the reduction in UACR was correlated with the changes in PBR confirms the role of the endothelial glycocalyx in the development of diabetic nephropathy and further supports the fact that the renal actions of GLP-1RAs and SGLT-2i do not depend solely on glycemic control but also on other direct mechanisms in the endothelium, such as increased NO production and the enhancement in chronic inflammation and oxidative stress [38]. The association between UACR and PWV/GLS ratio confirms the relationship between arterial stiffness and arterioventricular coupling, a fact that had been made evident only in the study by Smith et al., where UACR had significant predictive value for aortic PWV [39].

Both groups showed improvement in GLS in our study, although the magnitude of the improvement was almost three-fold higher in the combination group, and the results for the PWV/GLS ratio were similar. The effect of the GLP-1RAs+SGLT-2i combination on these indices has been shown only in a previous study by our group, where GLS and PWV/GLS were improved by 13% and 31%, respectively, after 12 months of treatment [12]. The effect of dulaglutide as monotherapy has been studied only by Basile et al. [40], in an observational study that lasted for only 6 months. As for dapagliflozin, studies by Tanaka et al. [41] and Xanthopoulos et al. [42] showed significant improvements after 6 and 12 months, respectively. In general, SGLT-2is have a protective effect on the myocardium and the mean improvement in GLS is approximately 17% [43]. This effect is mainly attributed to the reversion of cardiac remodeling, which is a process of inflammation and mitochondrial dysfunction leading, eventually, to cardiac hypertrophy and interstitial fibrosis. SGLT-2is revert this process by natriuresis, improvement in endothelial function, deactivation of the inflammasome, and increased ketogenesis, while they prevent the accumulation of extracellular matrices and myocardial fibrosis [44,45]. DPP-4is do not share such mechanisms, and their effect on GLS is generally neutral [46].

The beneficial effects of the combination treatment mark the significant clinical implications of our study. Although it is already known that GLP-1RAs and SGLT2-is are the first line of treatment in T2DM when there is overt cardiovascular disease or CKD, or when there is a high risk of CVD, such results extend the possible eligibility criteria for initiating these agents. Arterial stiffness and endothelial dysfunction are markers of subclinical vascular disease and, when available, they should be taken into account when evaluating a patient’s risk of adverse cardiovascular and renal events, even before prominent atherosclerosis or arterial hypertension become obvious. Such an aggressive approach could make a substantial difference in eventual comorbidities, thus offering remarkable benefits in terms of patient wellness and associated healthcare costs.

### Limitations

A major limitation of our study is the small study sample. In addition, the lack of a direct comparison between different DPP-4is should be noted as a limitation; patients in the DPP-4i group received any of the commercially available DPP-4is, so the results in this group are a class effect and cannot be attributed to a specific agent. However, the duration of our study is among the highest in relevant studies in the literature and, therefore, it is sufficient to investigate both the immediate and the sustained actions of the drugs studied. Prospective studies with larger sample sizes, a more prolonged duration of follow-up, and different combinations of GLP-1RAs and SGLT-2is are needed to extrapolate our results to a broader patient population.

## 5. Conclusions

Treatment with the dulaglutide and dapagliflozin combination for 12 months improved arterial stiffness, the endothelial glycocalyx, albuminuria, and myocardial function compared to DPP-4i, in patients with T2DM and albuminuria. These benefits are not dependent only on glycemic control, but also on the vascular and cardiac actions of these drugs. Therefore, early combination treatment has substantial cardiorenal benefits and should be an option for eligible patients, as it can contribute to the prevention of macro- and microvascular complications even at a sub-clinical level.

## Figures and Tables

**Table 1:** Baseline characteristics of the study population.

	All Patients (n = 60)	Dulaglutide + Dapagliflozin (n = 30)	DPP-4i (n = 30)	*p*-Value
Diabetes duration (y.)	9 [4,5,6,7,8,9,10,11,12,13,14,15,16,17]	8 [5,6,7,8,9,10,11,12,13]	10 ± 4	0.118
Age (y.)	61 ± 7	59 ± 7	63 ± 7	0.143
Sex (male/female), n (%)	(48/12), (80/20)	26/4 (87/13)	22/8 (73/27)	0.110
Risk factors				
LVEF, (%)	54 ± 10	56 ± 9	53 ± 8	0.210
Active smoking	(27/33) (45/55)	(14/16) (46/54)	(13/17) (43/57)	0.799
Arterial hypertension	(60/0) (100/0)	(30/0) (100/0)	(30/0) (100/0)	0.000
Dyslipidemia	(54/6) (90/10)	(28/2) (93/7)	(26/4) (87/13)	0.682
Family history of CAD	(25/35) (41/59)	(14/16) (46/54)	(11/19) (36/64)	0.419
eGFR, mL/min per 1.73 m^2^	92 ± 10	91 ± 9	93 ± 10	0.322
Cardiovascular medications, n (%)				
Beta-blockers	(20/40) (33/67)	(11/19) (36/64)	(9/21) (30/70)	0.669
CCBs	(21/39) (35/65)	(13/17) (43/57)	(8/22) (26/74)	0.176
ACEis or ARBs	(100/0) (100/0)	(30/0) (100/0)	(30/0) (100/0)	0.000
Diuretics	(16/44) (26/74)	(7/23) (23/77)	(9/21) (30/70)	0.559
MRAs	(7/53) (11/89)	(4/26) (13/87)	(3/27) (10/90)	0.688
Statins	(54/6) (90/10)	(28/2) (93/7)	(26/4) (86/14)	0.389

Scale variables are expressed as the mean ± standard deviation or median [interquartile range]. Nominal variables are presented as absolute and relative frequencies (%). LVEF: left ventricular ejection fraction; CAD: coronary artery disease; eGFR: estimated glomerular filtration rate; ACEis: angiotensin-converting enzyme inhibitors; ARBs: angiotensin receptor blockers; CCB: calcium channel blockers; MRAs: mineralocorticoid receptor antagonists; y: years.

**Table 2:** Changes in metabolic control.

Variable	Total (n = 60)	DPP4i (n = 30)	Dulaglutide + Dapagliflozin (n = 30)
HbA1C, %			
Baseline	8 ± 0.7	8.1 ± 0.8	7.9 ± 0.7
4 months	7.3 ± 0.7 ^†††^	7.5 ± 0.7 ^††^	7.2 ± 0.6 ^††^
Δ%	−8.7	−0.6	−0.7
12 months	7 ± 1 ^†††^	7.2 ± 0.7 ^†^	6.9 ± 0.6 ^††^
Δ%	−12.5	−0.9	−1.0
Fasting Blood Glucose, mg/dL			
Baseline	152 ± 42	157 ± 48	149 ± 45
4 months	129 ± 30 ^†††^	136 ± 44 ^††^	124 ± 27 ^††^
Δ%	−15.1	−13.3	−16.7
12 months	120 ± 31 ^†††^	123 ± 40 ^†††^	119 ± 24 ^†††^
Δ%	−21	−21.65	−20.9
Body Mass Index, kg/m^2^			
Baseline	32.7 ± 5	29.7 ± 6	35.2 ± 7
4 months	31.1 ± 3 ^†††^	29.4 ± 6	32.4 ± 5 ^†,^*
Δ%	−4.89	−1.01	−7.95
12 months	30.5 ± 4 ^†††^	29.3 ± 5	31.4 ± 4 ^†,^**
Δ%	−6.72	−1.34	−10.79

* Data are presented as the mean ± SD. Δ% indicates percentage change from baseline; BMI: body mass index; HbA1c: glycosylated hemoglobin; * *p* < 0.05, ** *p* < 0.01 for time × treatment interaction obtained via repeated-measures ANOVA. ^†^ *p* < 0.05, ^††^ *p* < 0.01, ^†††^ *p* < 0.001 for comparisons of 4 or 12 months vs. baseline via ANOVA using post hoc analysis with Bonferroni correction.

**Table 3:** Changes in renal function.

Variable	Total (n = 60)	DPP4i (n = 30)	Dulaglutide + Dapagliflozin (n = 30)
eGFR, mL/min per 1.73 m^2^			
Baseline	92 ± 10	91 ± 9	93 ± 10
4 months	91 ± 9	90 ± 6	95 ± 8 *
Δ%	−1.1	−1.1	2.1
12 months	91 ± 8	88 ± 9	97 ± 6 ^††,^**
Δ%	−1.1	−3.3	4.3
ACR, mg/g			
Baseline	335 ± 57	345 ± 48	326 ± 61
4 months	256 ± 49 ^††^	306 ± 60 ^†^	207 ± 55 ^††,^*
Δ%	−23.6	−11,4	−33.7
12 months	218 ± 40 ^†††^	295 ± 51 ^††^	142 ± 47 ^†††,^**
Δ%	−35	−14.5	−52.3

* DPP-4i: dipeptidyl peptidase 4 inhibitors; eGFR: estimated glomerular filtration rate. * *p* < 0.05, ** *p* < 0.01 for time × treatment interaction obtained via repeated-measures ANOVA ^†^ *p* < 0.05, ^††^ *p* < 0.01, ^†††^ *p* < 0.001 for comparisons of 4 or 12 months vs. baseline via ANOVA using post hoc analysis with Bonferroni correction.

**Table 4:** Changes in endothelial glycocalyx thickness and arterial stiffness markers.

Variable	Total (n = 60)	DPP4i (n = 30)	Dulaglutide + Dapagliflozin (n = 30)
PBR, 5–25 μm			
Baseline	2.11 ± 0.31	2.11 ± 0.31	2.10 ± 0.31
4 months	2.08 ± 0.29	2.16 ± 0.32	2.02 ± 0.26 ^†,^*
Δ%	−1.42	2.37	−3.81
12 months	2 ± 0.25 ^††^	2.08 ± 0.28	1.93 ± 0.23 ^††,^**
Δ%	−5.21	−1.42	−8.10
PWV, m/s			
Baseline	11.26 ± 2.45	10.64 ± 2.44	11.77 ± 2.37
4 months	10.75 ± 2.27	10.61 ± 2.55	10.89 ± 2.10 ^†,^**
Δ%	−4.6	−0.3	−7.48
12 months	10.62 ± 2.52	10.54 ± 2.84	10.70 ± 2.29 ^††,^***
Δ%	−5.7	−0.9	−9.1
SBP, mmHg			
Baseline	135.50 ± 19.80	131.13 ± 14.17	139.58 ± 23.17
4 months	135.17 ± 16.17	135.36 ± 15.80 ^†^	135 ± 16.74 ^†,^**
Δ%	−0.24	3.22	−3.28
12 months	132.15 ± 15.46	133.10 ± 12.51	131.30 ± 17.87 ^††,^**
Δ%	−2.47	1.50	−5.93
DBP, mmHg			
Baseline	83.32 ± 11.25	81.47 ± 11.30	85 ± 11.12
4 months	80.96 ± 10.40 ^†^	81 ± 10.40	80.93 ± 10.57 ^††^
Δ%	−2.83	−0.57	−4.78
12 months	79.23 ± 9.75 ^††^	78.33 ± 10.27 ^††^	80.06 ± 9.34 ^†††^
Δ%	−4.91	−3.85	−5.81
Central SBP, mmHg			
Baseline	126.84 ± 18.80	123.13 ± 11.66	130.21 ± 17.23
4 months	126.25 ± 14.20	124.46 ± 14.10	127.42 ± 14.41
Δ%	−0.46	1.08	−2.14
12 months	124.20 ± 16.18	125.13 ± 13.56	123.36 ± 18.42 ^†,^*
Δ%	−2.08	1.62	−5.26

* Data are presented as the mean ± SD or median (first quartile to third quartile). Δ% indicates percentage change from baseline; DBP, diastolic blood pressure; PBR, perfused boundary region; PWV, pulse wave velocity; SBP, systolic blood pressure; DPP-4i, dipeptidyl peptidase 4 inhibitors. * *p* < 0.05, ** *p* < 0.01, *** *p* < 0.001 for time × treatment interaction obtained via repeated-measures ANOVA. ^†^ *p* < 0.05, ^††^ *p* < 0.01, ^†††^ *p* < 0.001 for comparisons of 4- or 12-months vs. baseline via ANOVA using post hoc analysis with Bonferroni correction.

**Table 5:** Changes in LV myocardial deformation.

Variable	Total (n = 60)	DPP4i (n = 30)	Dulaglutide + Dapagliflozin (n = 30)
GLS, %			
Baseline	−17.89 ± 3.92	−18.45 ± 3.38	−17.42 ± 4.38
4 months	−18.50 ± 4.13 ^†^	−19.06 ± 3.45	−18.02 ± 4.67
Δ%	3.40	3.30	3.44
12 months	−20.03 ± 3.68 ^††^	−19.56 ± 3.85 ^†^	−20.59 ± 3.51 ^†††^
Δ%	11.96	6.01	18.19
PWV/GLS			
Baseline	−0.68 ± 0.15	−0.64 ± 0.17	−0.71 ± 0.24
4 months	−0.64 ± 0.20 ^†^	−0.63 ± 0.16	−0.64 ± 0.18 ^†^
Δ%	5.88	1.56	9.86
12 months	−0.56 ± 0.15 ^††^	−0.58 ± 0.16 ^†^	−0.54 ± 0.13 ^†††^
Δ%	17.64	9.37	23.94

Data are presented as the mean ± SD. Δ% indicates the percentage change from baseline. GLS, global longitudinal strain; PWV, pulse wave velocity; DPP-4i, dipeptidyl peptidase 4 inhibitors. ^†^ *p* < 0.05, ^††^ *p* < 0.01, ^†††^ *p* < 0.001 for comparisons of 4 or 12 months vs. baseline via ANOVA using post hoc analysis with Bonferroni correction.

## Data Availability

The datasets used and/or analyzed during the current study are available from the corresponding author upon reasonable request.
